# PB.40: Clinical recall audit: are we following national guidelines?

**DOI:** 10.1186/bcr3540

**Published:** 2013-11-08

**Authors:** F Kilburn-Toppin, S Karia, M Wallis

**Affiliations:** 1 Addenbrooke's Hospital, Cambridge, UK

## Introduction

Women with normal mammograms may be recalled from the NHSBSP if they indicate significant clinical symptoms at the time of screening. It is important that mechanisms are in place to record clinical abnormalities, with a clear written protocol.

## Methods

The Cambridgeshire screening population was examined from April 2009 to April 2012. Our standard was that all patients with clinical symptoms should have a clinical alert on NBSS, in keeping with 2005 assessment guidelines [1]. We analysed the number of women who were clinically recalled for this period, if a clinical alert was entered on NBSS, the symptoms underlying the clinical recall, and the clinical outcome.

## Results

A total of 50,785 patients were screened, of which 165 with normal mammography were placed on clinical recall. In total, 151 (92%) had a clinical alert on NBSS. Thirty-seven of these had an abnormality detected, of which the majority (*n *= 17) were cysts. Four (2% or 0.8 per 10,000 screened) had an invasive malignancy. A palpable lump was the commonest clinical symptom for recall, but also included nipple discharge, pain and patient anxiety.

## Conclusion

Electronic NBSS documentation for clinical recall is an important measure to ensure patients with normal mammograms but clinically significant symptoms are not returned to routine screening. Radiographers should receive appropriate training to recognise significant breast symptoms, with a clear written protocol for clinical recall. New local guidelines have been implemented prior to re-audit.
